# Survey on *Chlamydiaceae* in cloacal swabs from Swiss turkeys demonstrates absence of *Chlamydia psittaci* and low occurrence of *Chlamydia gallinacean*

**DOI:** 10.1371/journal.pone.0226091

**Published:** 2019-12-10

**Authors:** Barbara Renate Vogler, Michal Trinkler, Hanna Marti, Nicole Borel, Theresa Pesch, Barbara Prähauser, Richard Hoop, Prisca Mattmann, Sarah Albini

**Affiliations:** 1 National Reference Centre for Poultry and Rabbit Diseases (NRGK), Institute for Food Safety and Hygiene, Vetsuisse Faculty, University of Zurich, Zurich, Switzerland; 2 Institute for Veterinary Pathology, Vetsuisse Faculty, University of Zurich, Zurich, Switzerland; Tokat Gaziosmanpasa University, TURKEY

## Abstract

In Switzerland, domestic turkey meat is a niche product. Turkeys are fattened on mixed family-based farms scattered across the country, with most providing access to an uncovered outdoor pasture for the birds. Swiss fattening turkeys may therefore get infected with *Chlamydiaceae* via wild birds or their faeces, potentially shedding these bacteria at a later stage. The aim of the present study was to acquire baseline data about the shedding of *Chlamydiaceae* in clinically unremarkable Swiss fattening turkeys at slaughter, potentially exposing slaughterhouse workers to infection. In this large-scale study, 1008 cloacal swabs of Swiss turkeys out of 53 flocks from 28 different grow-out farms with uncovered outdoor pasture were collected over the course of 14 months and examined for the occurrence of *Chlamydiaceae* by a family-specific 23S-rRNA real-time PCR. Positive samples were further analyzed by *Chlamydia psittaci* (*C*. *psittaci*)-specific real-time PCR and the Arraymate DNA Microarray for species identification. All samples were negative for *C*. *psittaci*, but seven swabs out of one flock were tested positive for *Chlamydia gallinacea* (0.7%). Although turkeys with access to pasture may have contact with *Chlamydiaceae*-harbouring wild birds or their faeces, the infection rate in Swiss turkeys was shown to be low.

## Introduction

Members of the family *Chlamydiaceae* are obligate intracellular Gram-negative bacteria with a biphasic life cycle [1]. *Chlamydia psittaci* (*C*. *psittaci*, formerly also known as *Chlamydophila psittaci*) is a zoonotic agent causing chlamydiosis in birds and humans. Chlamydiosis is also termed psittacosis in psittacine birds or ornithosis in other bird species and humans. The infection route is oral, by inhalation of feather dust or aerosolized faecal material [2]. The species *C*. *psittaci* is divided into 13 genotypes (A—F, E/B, and others), based on the DNA sequence of the outer membrane protein A (OmpA) [3]. The different genotypes have a certain degree of host specificity, but they can also infect other species. For example, genotypes D, F and E/B have been found in turkeys, but they can also infect other avian species as well as humans [4]. All avian strains have a zoonotic potential and humans exposed to *C*. *psittaci*-positive birds have a substantial risk of becoming infected [1, 5]. Human infections are commonly traced to psittacines [6], but ornithosis can also occur in persons with regular contact to poultry, such as slaughterhouse workers and veterinarians [4, 7, 8].

*Chlamydia psittaci* infections are abundant in European chicken and turkey flocks as demonstrated by studies from Belgium and France [2, 9–11], the Netherlands [12], and Germany [13–15]. Diseased flocks exhibited respiratory diseases, e.g. nasal and ocular discharge, sinusitis, lower respiratory tract infection and increased morbidity and mortality during rearing. Another *Chlamydia* species, previously known as “atypical chicken chlamydia” (ACC) and later named *Chlamydia gallinacea* (*C*. *gallinacea*) [16], has been found in cloacal swabs or faeces from clinically healthy chickens from various European countries, e.g. Germany, France, Greece, the Netherlands, Slovenia and Croatia [8, 17, 18, 19], and it was also detected in turkeys [18, 20]. Subsequently, *C*. *gallinacea* was shown to be an endemic chlamydial species in chickens [19, 21–22]. A report of atypical non-*C*. *psittaci* pneumonia in poultry slaughterhouse workers suggested that *C*. *gallinacea* has a zoonotic potential [17].

In Switzerland, 14.6% of the consumed turkey meat is produced locally, yielding approximately 1’742 tons of slaughter weight in 2016 [23]. The overall animal health is very good, and respiratory problems, such as mycotic (aspergillosis), viral or bacterial infections, are only seen in individual animals. A search in the necropsy case data base of the National Reference Laboratory of the last 30 years could not detect a single entry regarding a *C*. *psittaci* positive case in commercial poultry, but comprehensive data on the flock level is missing.

Due to the market share of 90% in domestic turkey meat (2016), accounting to approximately 161,400 turkeys slaughtered per year, the vertical integration “company A” is the representative example for the Swiss turkey production. In this vertical integration chain, the hatchery and their associated rearing farms supply the contract grow-out farms with either one day-old poults or six week old birds. The grow-out farms are scattered across the country (cantons of Berne, Fribourg, Vaud, Aargau, St. Gallen, Thurgau and Zurich) and keep approximately 2000 animals with 2 ½—3 ½ turnovers each year. All of the birds are raised in a subsidized pasture setting according to Swiss Law (so called “BTS” = particularly animal-friendly housing systems and “RAUS” = open-air programme for animals) [24]. Bedding material is stored indoors in a hygienic way and expanded feed is used. Antibiotic grow-promoters are prohibited since 1999 [25]. Maximum stocking density is 36.5 kg / m^2^, as regulated by the Swiss Animal Welfare Law [26]. Males and females are always kept in separate compartments of a stable with a corresponding winter garden (an enclosed area where birds have access to fresh air and sunlight, comparable to an aviary), thus a flock consists of animals of the same gender. At six to seven weeks of age, the turkeys get access to a fenced uncovered outdoor pasture area (RAUS regulation as mentioned above). At slaughtering age (females at 11 to 12 weeks and males at 15 to 20 weeks of age), the turkeys are transported from the grow-out farms to the abattoir of “company A” in the canton of Thurgau, Eastern Switzerland.

Turkeys raised with outdoors access to pastures potentially have contact to *Chlamydiaceae*-harbouring wild birds or their faeces. At slaughter, the reception area mainly gets soiled by faeces of arriving animals, thus it is of interest, whether shedding of *Chlamydiaceae* occurs at this timepoint, potentially exposing the personnel working in the turkey reception area. Therefore, despite the apparent absence of health problems in turkey flocks, farmers and slaughterhouse workers, the current study aimed to acquire baseline data about the shedding of *Chlamydiaceae* in clinically unremarkable Swiss fattening turkeys at slaughter.

## Materials and methods

### Cloacal swab samples of Swiss turkeys

To allow for resource effective sampling in this large-scale study, the sample collection was centralized without the need for extra manpower. Thus, collection of cloacal swabs using dry flocked swabs (FLOQ Swabs^®^, Copan Italia, Brescia, Italy) at the Swiss turkey abattoir of “Company A” in the canton of Thurgau was chosen, because swabs could be i) taken by the personnel of the abattoir on the shackle line after stunning and sticking; ii) stored on site at 4° C for up to 4 days and sent to the laboratory in a small next day standard parcel, and iii) frozen at—20° C upon arrival at the laboratory.

It was aimed to analyze at least 1000 swabs (>0.5% of slaughtered turkeys) with 20 swabs per flock to represent all cantons and grow-out farms. In total, 1008 cloacal swabs from 53 clinically unremarkable turkey flocks from 28 different grow-out farms of the vertical integration “company A” were collected over the course of 15 months (April 2016 until June 2017; Table 1). Turkey health is considered excellent on the farms of “company A”, with only two cases of histomoniasis in the past 20 years and no record of chlamydial infections. None of the turkeys did present any clinical signs at slaughter and no respiratory disease or elevated mortality had been noticed in these turkey flocks prior to slaughter. There were no health issues concerning the farmers or the personnel of the abattoir. Workers and visitors have to declare that they are not experiencing any health problems at the entry of the abattoir building.

**Table 1:** Number of cloacal swab samples from Swiss turkey farms located in seven different cantons. Farm-ID consists of the canton where the farm is located followed by a consecutive number.

Region	Farm-ID (flock number)	Number of samples per flock
Western Switzerland	Berne-1 (1)	20
	Berne-2 (1; 2; 3)	19; 20; 20
	Fribourg-1 (1)	20
	Fribourg-2 (1; 2))	20; 20
	Fribourg-3 (1; 2)	20; 20
	Fribourg-4 (1)	20
	Fribourg-5 (1)	20
	Fribourg-6 (1)	20
	Fribourg-7 (1; 2)	10; 20
	Fribourg-8 (1; 2)	10; 21
	Fribourg-9 (1; 2)	20; 20
	Fribourg-10 (1; 2)	20; 20
	Fribourg-11 (1;2)	20; 20
	Vaud-1 (1; 2)	4; 20
	Vaud-2 (1; 2)	20; 20
Eastern Switzerland	Aargau-1 (1)	20
	Aargau-2 (1; 2)	20; 20
	St. Gallen-1 (1)	20
	St. Gallen-2 (1; 2)	20; 20
	St. Gallen-3 (1; 2; 3)	21; 20; 20
	St. Gallen-4 (1; 2; 3)	5; 20; 20
	St. Gallen-5 (1; 2)	20; 20
	Thurgau-1 (1; 2)	20; 20
	Thurgau-2 (1; 2; 3)	20; 20; 20
	Thurgau-3 (1)	20
	Thurgau-4 (1; 2; 3)	20; 20, 20
	Zurich-1 (1)	20
	Zurich-2 (1; 2; 3)	20; 18; 20
Total	28 farms / 53 flocks	1008

### DNA extraction from cloacal swab samples

DNA from cloacal swabs was extracted with the Maxwell^®^ Buccal Swab LEV DNA Purification kit (Promega, Madison, USA), with an elution volume of 100 μl, using the Maxwell^®^ 16 (Promega), as previously performed in a *Chlamydiaceae* abattoir study in swine [27]. Extracted DNA was measured on a Nanodrop 2000c spectrophotometer (Thermo Fisher Scientific, Waltham, MA, USA) to determine DNA quantity and quality (260/280 value) and stored at—20° C until further analysis.

### Detection and identification of *Chlamydiaceae* by various assays

**Fig 1:**
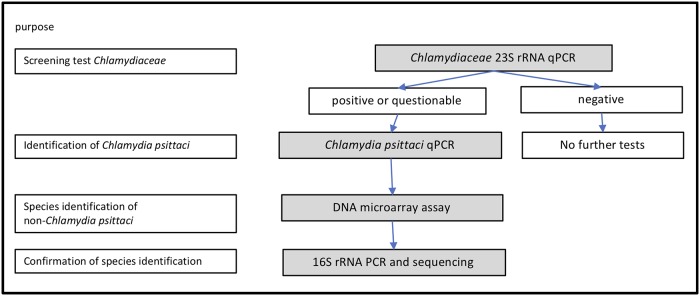
Hierarchical approach for the detection and identification of *Chlamydiaceae* in cloacal swab samples from turkeys (qPCR = quantitative PCR).

In line with the recommendations of the terrestrial manual of the OIE for the diagnosis of avian chlamydiosis [28], a hierarchical approach was applied for the detection and identification of *Chlamydiaceae* (Fig 1).

The family specific 23S rRNA quantitative PCR (qPCR) for the detection of *Chlamydiaceae* was used as a screening test. Positive or questionable samples were analyzed with a *Chlamydia psittaci* qPCR. Non-*Chlamydia psittaci* samples were specified with a DNA microarray assay and the results were corroborated by 16S rRNA PCR and sequencing.

#### Quantitative PCR (qPCR) for the detection of *Chlamydiaceae*

All samples (n = 1008) were analyzed on an Applied Biosystems^™^ 7500 Real-Time PCR System (Thermo Fisher Scientific, Waltham, MA, USA) with the fast cycling profile, based on the 23S rRNA *Chlamydiaceae* family-specific real-time PCR as described previously [29], with primers Ch23S-F (5'-CTGAAACCAGTAGCTTATAAGCGGT-3'), Ch23S-R (5'-ACCTCGCCGTTTAACTTAACTCC-3') and probe Ch23S-P (5’-FAM-CTCATCATGCAAAAGGCACGCCG-TAMRA-3’), modified [30] to include an internal positive control (enhanced green fluorescent protein (eGFP); [31]), with primers EGFP-1-F (5’-GACCACTACCAGCAGAACAC-3’), EGFP-10-R (3’-CTTGTACAGCTCGTCCATGC-5’) and probe EGFP1-P (5’-HEX-AGCACCCAGTCCGCCCTGAGCA-BHQ-1-3’). Primers and probes were purchased from Microsynth, Balgach, Switzerland. Cycling conditions were 95°C for 20 s, followed by 45 cycles of 95°C for 3 s and 60°C for 30 s. Amplicon sizes are 111 base pairs (bp) for the *Chlamydiaceae* quantitative real-time PCR (qPCR) and 176 bp for the eGFP qPCR. In each run, three negative controls (aqua bidest.) were included. A sevenfold dilution series of *C*. *abortus* DNA was included in each run both as a positive control and as a standard curve. All samples were tested in duplicate and the cycle threshold was set at 0.1 in each run. Samples were interpreted as positive if the mean cycle threshold (Ct value) was < 38. Quantitation of positive samples was done by creating a standard curve of the above mentioned *C*. *abortus* dilution series with the software of the Applied Biosystems^™^ 7500 Real-Time PCR System. The *Chlamydiaceae* copy number for each sample is directly calculated by the software. As *C*. *abortus* and *C*. *gallinacea* each harbour one copy of the 23S rRNA gene, the number of *C*. *gallinacea* copies can be directly calculated from the standard curve.

#### qPCR for the detection of *Chlamydia psittaci*

Samples positive in the *Chlamydiaceae* qPCR were tested in duplicate with a *C*. *psittaci*-specific qPCR targeting the *ompA* gene (76 bp amplicon size) [32], including an internal eGFP-control as described elsewhere [33]. Samples (n = 7) were analyzed on an Applied Biosystems^™^ 7500 Real-Time PCR System (Thermo Fisher Scientific) with the fast cycling profile, with primers CppsOMP1-F (5'-CACTATGTGGGAAGGTGCTTCA-3'), CppsOMP1-R (5'-CTGCGCGGATGCTAATGG -3') and the minor groove binding probe CppsOMP1-P (5’MGB-FAM-CGCTACTTGGTGTGAC-TAMRA-3’), primers EGFP-1-F (5’-GACCACTACCAGCAGAACAC-3’), EGFP-2-R (5’-GAACTCCAGCAGGACCATG-3’) and the probe EGFP1-P (5’-HEX-AGCACCCAGTCCGCCCTGAGCA-BHQ1-3’). Primers and probes were purchased from Microsynth, Balgach, Switzerland. Cycling conditions were 2 min at 50° C, 10 min at 95° C, followed by 40 cycles of denaturation at 95° C for 15 sec and annealing at 60°C for 1 min. A negative control (aqua bidest.) and a positive control (synthesized oligonucleotide of the *ompA* gene of a *C*. *psittaci* field isolate “T0592/03, amazon parrot”; purchased from Microsynth) were used in duplicates in each run. This PCR was carried out based on the experience gained during a recent study [34], where samples with a very high Ct-value in the *Chlamydiaceae* qPCR could be identified with the *C*. *psittaci* qPCR, but neither with the less sensitive Microarray assay nor the 16S rRNA PCR and sequencing.

#### Arraymate microarray for species identification of *Chlamydiaceae*

Samples positive by *Chlamydiaceae*-qPCR, but negative in the *C*. *psittaci* qPCR, were further processed with the “ChlamType-23S AS-4 Kit” (Abbott, Chicago, Illinois, USA; formerly Alere Technologies GmbH), a species-specific 23S rRNA Arraymate Microarray yielding a 171 bp product, as described before [27, 35]. Briefly, the analysis by DNA microarray assay required the following steps: amplification and labelling of the sample DNA by conventional PCR followed by hybridization to specific DNA probes and staining. i) biotinylation PCR: the sample DNA was amplified and biotin labelled using the method as described previously [35]. The reaction mix contained 1 μl (< 150 ng/μl) sample DNA template, 1x PCR buffer with MgCl_2_ (Roche Diagnostics GmbH), 2.5 mM MgCl_2_ (Roche Diagnostics GmbH), 0.1 nM dNTP (PCR Nucleotide Mix, Roche Diagnostics GmbH), primers eGFP-11-F (5’-CAGCCACAACGTCTATATCATG-3’) and eGFP-10-R-Bio (5’- Bio-CTTGTACAGCTCGTCCATGC-3’), 1 μl eGFP template, primers U23F-19 (5’- ATTGAMAGGCGAWGAAGGA-3’) and 23R-22 (5’- biotin-GCYTACTAAGATGTTTCAGTTC-3’) (purchased from Microsynth) and 0.2 μl FastStart Taq DNA Polymerase (Roche Diagnostics GmbH). The total reaction volume was 20 μl. The cycle conditions were 96°C for 10 min, followed by 39 cycles of 94°C for 30 s, 50°C for 30 s and 72°C for 30 s and a last step of 72°C for 4 min. ii) hybridization: the labelled DNA was hybridized using the Hybridization Kit 245200100 (Alere Technologies GmbH) and analyzed using the ArrayStrip^™^ system (ChlamType-23S AS-4 Kit, Alere Technologies GmbH, Jena, Germany), as previously established [35]. The current version identifies eleven species of *Chlamydia* (*C*. *abortus*, *C*. *caviae*, *C*. *felis*, *C*. *psittaci*, *C*. *pecorum*, *C*. *pneumoniae*, *C*. *suis*, *C*. *muridarum*, *C*. *trachomatis*, *C*. *avium*, *C*. *gallinaceae*) and nine *Chlamydia*-like organisms (*Simkania*, *Waddlia*, *Protochlamydia aemoebophila*, *Proteochlamydia naegleriophila*, *Neochlamydia hartmannellae*, *Parachlamydia acanthamoebe*, *Criblamydia sequanensis*, *Chlamydiales xenoturbella*, *Estrella lausannensis*).

#### Conventional 16S rRNA gene PCR and sequencing

To confirm the identification of positive samples, the conventional 16S rRNA PCR was performed as described [36], using the primers 16S-IGF (5'-GATGAGGCATGCAAGTCGAACG-3') and 16S-IGR (5'-CCAGTGTTGGCGGTCAATCTCTC-3') to amplify a sequence of 298 bp. Per tube, 50 μl reaction mix was used, containing 1 μl (< 150 ng/μl) sample template, 1x PCR buffer with MgCl_2_ (Roche Diagnostics GmbH), 0.5 mM MgCl_2_ Stock Solution (Roche Diagnostics GmbH), 0.2 mM dNTP (PCR Nucleotide Mix, Roche Diagnostics GmbH), 300 nM of the primers 16S-IGF and 16S-IGR and 0.02 U/μl FastStart Taq DNA Polymerase. Cycling conditions were 95° C for 5 min, followed by 40 cycles of 95° C for 60 s, 65° C for 60 s and 72° C for 90 s and a final step of 72° C for 10 min. A negative control (aqua bidest.) and a positive control (DNA of *Chlamydia suis* S45/6 (type strain)) were used in each run. The PCR products were purified with the QIAquick PCR Purification Kit (Qiagen) according to the manufacturer’s instructions. The 298 bp product was Sanger sequenced by Microsynth. Comparsion of obtained sequences was done with the standard nucleotide Basic Local Alignment Search Tool (BLASTN) from the National Center for Biotechnology Information (NCBI). Sequence identity of the obtained sequences were compared to the nucleotide sequence of “*Chlamydia gallinacea* strain JX-1 chromosome, complete genome” (GenBank accession number: CP019792.1)

### Ethics statement

Since the study used cloacal swab samples of turkeys collected at slaughter (after stunning and sticking), no approval of the ethics committee was sought.

## Results

Out of 1008 cloacal swabs of turkeys tested, seven (0.7%) were positive in the *Chlamydiaceae* qPCR. All originated from flock 1 in farm Berne-1 and were collected in May 2017. These seven samples tested negative by the *C*. *psittaci* qPCR but were identified as *C*. *gallinacea* by Arraymate Microarray. This was corroborated by sequencing a 298 bp 16S rRNA gene fragment and subsequent BLASTN search against the NCBI databank, resulting in 100% nucleotide identity with *C*. *gallinacea* strain JX-1 (GenBank accession number: CP019792.1) in six of the sequences and 87.9% in one sample due to low sequence quality (GenBank accession numbers: MN056016–22) (Table 2). *Chlamydiaceae*-DNA content compared to total DNA content (*Chlamydia*-%) extracted from cloacal swabs was 0.0006% to 0.0074%, corresponding to 3.15 E +04 to 1.96 E +06 copies of the 23S rRNA gene per swab (Table 2). The sample with the lowest content yielded suboptimal sequencing data. Due to the perfect sequence match in the six other samples, the sample with poor sequence quality was not repeated.

**Table 2:** 23S rRNA *Chlamydiaceae* qPCR-positive cloacal swab samples of turkeys at slaughter originating from flock 1 in farm Berne-1.

sample	23S rRNA *Chlamydiaceae* qPCR		*ompA* gene *C*. *psittaci* qPCR	Arraymate Microarray	Conventional 16S rRNA gene PCR and sequencing		
	*Chlamydia*-%^1^	23S rRNA gene copies / swab^2^			Sequencing result	16S rRNA sequence identity [%]^3^	GenBank accession number
59–3	0.058	3.20E+05	negative	*C*. *gallinacea*	*C*. *gallinacea*	100	MN056016
59–4	0.0006	3.15E+04	negative	*C*. *gallinacea*	*C*. *gallinacea*	87.9	MN056017
59–8	0.014	1.29E+05	negative	*C*. *gallinacea*	*C*. *gallinacea*	100	MN056018
59–10	0.0066	3.87E+06	negative	*C*. *gallinacea*	*C*. *gallinacea*	100	MN056019
59–16	0.0074	1.96E+06	negative	*C*. *gallinacea*	*C*. *gallinacea*	100	MN056020
59–17	0.0014	2.73E+05	negative	*C*. *gallinacea*	*C*. *gallinacea*	100	MN056021
59–20	0.0045	3.13E+05	negative	*C*. *gallinacea*	*C*. *gallinacea*	100	MN056022

*C*. *psittaci = Chlamydia psittaci*; *C*. *gallinacea* = *Chlamydia gallinacea*

^1^*Chlamydiaceae*-DNA content compared to total DNA content

^2^The *Chlamydia gallinacea*-genome contains one copy of the 23S rRNA

^3^Compared to the nucleotide sequence of *Chlamydia gallinacea* strain JX-1 chromosome, complete genome (GenBank: CP019792.1)

## Discussion

The current study examined 1008 cloacal swab samples from Swiss fattening turkeys for the presence of *Chlamydiaceae*. Samples were collected continuously during all seasons between April 2016 and June 2017 at the abattoir. This corresponds to a sample size of more than 0.5% of all Swiss fattening turkeys slaughtered over this time period (n = 188’300).

While *C*. *psittaci* was not detected in any sample, seven swabs taken from one flock were positive for *C*. *gallinacea*. The observed infection rate (*C*. *gallinacea* positivity 0.7%) seems rather low, however, this data on *Chlamydiaceae* occurance cannot be directly compared to actual prevalence studies from other countries [19, 21].

In this study, turkeys were sampled by taking a single swab at slaughter in the abattoir of company A and not on the grow-out farms, because i) potential *Chlamydiaceae* infection might have accumulated over the whole live-span of the bird, ii) shedding in ready-to-slaughter turkeys is potentially increased due to transport stress [2], and iii) this setup allowed for resource effective sampling by centralizing it at the abattoir, with the help of the local personnel collecting the swabs, and sampling dead carcasses instead of live animals thereby bypassing the obligation to acquire an animal experimental permit. The restriction of this setup was, however, that only one sample per turkey could be taken due to limited time resources of the personnel. As the study aimed at collecting baseline data on shedding of *Chlamydiaceae* in turkeys at slaughter and the contamination of the abattoir reception area is mainly due to faeces (transport boxes etc), cloacal swabs were chosen to measure faecal shedding. In diseased birds, *C*. *psittaci* DNA can be found in sinuses, air sacs, lung and liver and various swab samples [1], however, in asymptomatically infected poultry, DNA of *C*. *psittaci* and other Chlamydia species can be found in oral or nasopharyngeal, conjunctival and cloacal swabs or faecal samples [8, 19, 21,]. Although testing oral swabs for *Chlamydiaceae* was shown to result in a higher positivity rate than cloacal swabs in healthy chicken, ducks, geese and pigeons [21], cloacal swabs were chosen in the current study. Reasons for this decision are i) the fact that contamination of the reception area is mainly due to faeces, ii) the ease of taking samples on the shackle line by the abattoir personnel, and iii) experiences from earlier studies [19, 34], where shedding of chlamydiae via the intestinal tract had been demonstrated in faecal samples and cloacal swabs. Shedding via the intestinal tract is especially important for *C*. *gallinacea* where the highest load of average bacterial genome number was found in cloacal swabs, followed by oral swabs, lung, heart, kidney, liver and other organs [21]. Moreover, despite intermittent *Chlamydiaceae* shedding [37], a recent study on 205 healthy pigeons culled within a population management programme [34] using paired samples of cloacal swabs and liver detected paired samples to be positive for *Chlamydiaceae* (*C*.*psittaci* or *C*. *avium*) in 19 pigeons, and positive cloacal swabs alone in another 27 animals; whereas only two pigeons were positive only in the liver.

The detection of *Chlamydiaceae* shedding in healthy flocks is important because it was observed earlier [2] that the slaughterhouse workers in the poultry reception area in abattoirs are endangered of becoming infectedthrough feather dust or faecal material. This observation may be explained through increased chlamydial shedding from live animals that are stressed by transportation and crowding, resulting in a greater contamination of the environment. Although the infection rate was very low (*C*. *gallinacea* positivity of 0.7%) in the current study, it cannot be ruled out, that one high shedding flock or few high shedding animals may contaminate the reception area and pose a threat to human abattoir workers.

To date, the zoonotic potential originating from *C*. *gallinacea* infections is unclear [1]. *C*. *gallinacea* was shown to be excreted by healthy chicken flocks in the production phase [19, 22] or at slaughter [17, 18] and is also present in turkeys [20]. A possible link between atypical pneumonia in slaughterhouse workers and *C*. *gallinacea* was suspected in a report from France [17], where only *C*. *gallinacea* but not *C*. *psittaci* was detected in cloacal swabs of 13/25 chicken flocks. However, microbiological confirmation was not performed in the human patients and thus it could not be confirmed that *C*. *gallinacea* was involved in these human pneumonia cases. Moreover, bird-to-human transmission could not be demonstrated in a recent study on farmers keeping *C*. *gallinacea*-positive chicken flocks [19]. Further studies are needed to prove or exclude the zoonotic potential of *C*. *gallinacea*. The present data and the absence of health issues in both, the turkey farmers and the abattoir workers from “company A” does currently not warrant a survey in humans.

Although all Swiss turkeys produced by “company A” have outdoor access with recurrent contact to *Chlamydiaceae*-reservoir species like pigeons or other wild birds [38–40], pasture production alone does not seem to endanger turkeys for chlamydial infections. Other factors such as low number of animals per farm, low density of animals per stable, expanded feed, good on-farm hygiene management with hygienic storage of bedding material, and health monitoring of the animals seem to be important factors contributing to raising *Chlamydiaceae*-free turkeys.
